# Supplementary material for: Determination of Pesticide Residues in IV Range Artichoke (Cynara cardunculus L.) and Its Industrial Wastes
Source: Foods. 2023 Apr 26;12(9):1807. doi: 10.3390/foods12091807 (PMC10178484; doi:10.3390/foods12091807)
Supplement: Supplementary file 1 [file foods-12-01807-s001.zip › foods-2299031-supplementary.pdf]

Table S1. Pesticide characteristics, MRM, and validation parameters.

Pesticide	Type[y]	Precursor	Product	Intercept	Slope	r^2	MRL	LOQ	Apparent recovery (mg/kg)			RSD_r	RSD_{wR}	ME
		ion (m/z)	ion (m/z)						0.005	0.025	0.25	LOQ		%
									% ± RSD%			%RSD		
Acephate	I	183.9	143.0	3525.369	5921281.59	0.9997	0.01	0.005	86.5±3.1	78.7±6.3	75.8±1.3	6.2	4.4	1.89
**Acetamiprid***	I	223.0	126.1	156982.1	10488947.4	0.9900	0.7	0.025	27.1±6.3	94.0±3.5	90.2±1.0	3.9	6.6	3.05
Aldicarb	N	208.0	116.0	3550.277	2708849.52	0.9992	0.02	0.025	27.8±6.6	100.2±7.9	92.2±2.2	6.9	4.7	-7.03
Aldicarb fragment	N	116.0	89.1	11016.16	5609980.02	0.9996		0.005	111.5±1.5	92.3±5.6	93.3±4.2	4.3	17.7	-5.28
Aminocarb	I	209,1	137.2	-49707.85	32033195.5	1.0000		0.005	98.6±3.9	88.9±4.4	83.9±1.0	4.6	4.1	1.24
Avermectin B1a	A	890.5	305.1	-20.40678	10337.0599	0.9995	0.01	0.25	125.0±5.0	66.4±3.6	81.4±5.1	3.6	11.2	-7.02
Azaconazole	F	300.0	158.9	13440.75	5957637.71	0.9995		0.005	79.4±9.5	90.4±6.2	90.4±1.1	4.4	5.7	0.53
Azamethiphos	I	325.0	182.9	16884.02	9430679.47	0.9995		0.025	12.3±10.5	95.2±3.1	97.4±2.5	3.7	4.6	-21.48
Azinphos-ethyl	I	346.1	132.0	7970.237	1240286.71	0.9996	0.02	0.025	69.9±13.0	85.4±7.6	98.9±2.6	5.4	6.1	-3.11
Azinphos-methyl	I	318.0	132.1	11562.27	1554791.44	0.9953	0.01	0.005	96.4±8.7	87.9±8.6	90.9±4.4	7.1	8.1	-4.50
**Azoxystrobin***	F	404.0	372.2	57243.40	19527396.7	0.9986	5.00	0.005	100.9±0.6	85.8±0.5	84.3±2.67	1.2	4.8	-0.81
Beflubutamid	H	356.0	91.0	94952.25	11332484.4	0.9972	0.02	0.005	90.5±0.9	87.7±3.1	90.9±1.6	5.4	6.4	-0.91
Benalaxyl	F	326.1	148.1	104927.9	12471271.7	0.9946	0.01	0.005	96.5±2.9	93.2±2.1	87.1±2.8	3.3	8.2	0.95
Benzoximate	A	364.1	198.9	73957.59	9967676.75	0.9959		0.005	98.5±4.8	75.4±3.3	80.5±2.0	4.2	5.4	-3.58
Bitertanol	F	338.1	70.1	-1102.308	458374.814	0.9999	0.01	0.005	113.7±11.9	90.3±7.9	79.1±5.2	5.9	9.0	2.30
**Boscalid***	F	343.0	307.1	110.2065	1247341.92	1.0000	5.00	0.005	79.6±11.0	88.5±1.2	90.8±3.9	2.0	5.6	3.14
Bromuconazole	F	377.9	159.0	-170.0283	698117.207	0.9995	0.01	0.005	106.3±13.0	100.4±6.2	88.2±0.3	5.8	18.9	1.37
Bupirimate	F	317.1	166.1	18038.71	4376927.70	0.9991	0.01	0.005	85.2±6.7	90.3±2.5	89.3±2.5	2.6	18.8	-0.61
Buprofezin	I	306.1	201.2	40616.97	8360338.9	0.9978	0.01	0.005	93.7±2.8	90.6±2.5	86.6±2.5	1.8	6.3	-1.98
Butocarboxim	I	208.0	116.0	11016.16	5609980.02	0.9996		0.025	61.2±1.3	92.3±5.6	93.3±4.2	4.3	17.7	-5.28
Carbaryl	I	202.0	145.0	9876.210	3462149.13	0.9988	0.01	0.005	78.0±1.4	92.1±0.8	94.2±1.0	3.3	8.7	-0.16
Carbendazim	F	192.0	160.1	141802.1	32981587.5	0.9984	0.1	0.025	36.3±5.6	86.5±2.8	80.9±0.5	3.0	5.3	-2.28
Carbofuran	I	222.1	165.1	91581.33	24466005.1	0.9985	0.00	0.005	101.3±3.9	110.8±1.4	107.2±1.1	2.0	6.7	-5.07
Carboxin	F	236.0	143.0	54867.36	13001038.8	0.9983	0.03	0.005	94.1±3.5	73.9±5.5	71.9±1.6	7.2	3.7	-14.05
Carfentrazone-ethyl	H	412.0	366.1	11175.38	1663675.94	0.9977	0.02	0.005	93.7±6.5	82.1±3.4	83.6±2.6	2.8	5.9	0.96
**Chlorantraniliprole***	I	483.9	452.9	972.9416	1177606.05	0.9996	2.00	0.005	91.7±13.5	80.4±0.2	81.5±1.8	0.4	7.7	2.93
Chlorfenvinphos	I	359.0	155.1	14085.74	3490444.20	0.9991	0.01	0.005	92.7±4.2	94.6±2.1	90.9±0.8	1.6	20.2	-0.25
Chloridazon	H	222.0	77.1	24974.90	4961903.38	0.9979		0.025	54.1±3.5	93.2±3.6	87.4±0.4	4.6	5.1	-7.13
Chlorotoluron	H	213.1	72.0	70745.55	14683833.2	0.9983	0.01	0.005	88.3±2.8	93.4±1.2	90.4±1.4	1.1	4.7	-2.19
Chloroxuron	H	291.0	72.1	78134.64	11656079.5	0.9966	0.01	0.005	89.7±3.8	87.8±2.2	90.0±2.1	3.0	5.1	-3.67
Chlorpyriphos	I	349.9	197.9	1134.977	1419134.09	0.9996		0.005	118.1±10.9	96.4±0.4	86.8±2.5	0.5	11.0	-0.10
Chlorpyriphos-methyl	I	322.0	125.0	572.5789	512605.279	0.9991		0.005	97.5±4.2	103.0±5.8	87.0±1.2	9.7	12.8	3.73
Clofentezin	I	303.0	138.0	38445.34	4268225.02	0.9959	0.02	0.005	87.7±7.0	90.2±0.3	88.4±1.8	0.8	14.6	-0.54
Clomazone	H	240.0	125.1	20195.09	12231387	0.9993	0.01	0.005	84.4±4.8	94.0±3.3	90.3±1.4	2.6	4.5	1.17
Coumaphos	I	363.0	226.9	31595.87	4332937.91	0.9976		0.005	106.5±3.5	93.3±1.6	89.9±3.3	3.8	6.6	2.89
Cyazofamid	F	325.0	108.1	65547.05	7513515.42	0.9960	0.01	0.005	82.9±5.1	77.4±3.3	83.0±1.5	2.8	4.7	-2.47
Cycloate	H	216.1	55.0	7287.719	1398843.34	0.9967		0.005	96.7±8.8	97.7±0.4	88.5±1.4	0.3	9.3	-2.11
Cycluron	H	199.2	72.1	90208.30	12046405.8	0.9971		0.005	89.7±3.5	90.8±2.1	91.2±2.2	1.7	5.4	-1.65
Cymiazole	F	219.0	144.0	2493.620	2434379.08	0.9994		0.25	49.3±36.9	68.5±2.5	102.8±1.5	2.9	6.9	-35.69
**Cymoxanil***	F	199.0	128.1	21094.72	2518734.41	0.9947	0.01	0.005	74.3±8.5	83.9±8.1	89.2±0.8	5.7	4.2	-5.83
Cyproconazole	F	292.1	70.1	16448.65	3032963.25	0.9979	0.1	0.005	94.4±13.6	89.2±1.9	88.2±1.7	2.7	11.0	0.88
Cyprodinil	F	226.1	93.1	16844.78	5160815.53	0.9992	4	0.005	97.0±8.8	89.5±6.1	90.2±1.4	4.6	5.1	0.00
DEET	I	192.1	119.0	194151.9	30472163.8	0.9978		0.005	88.1±1.7	93.0±0.4	91.3±1.0	1.9	8.6	0.56
Desmedipham	H	318.1	182.2	8509.233	5280021.35	0.9927	0.01	0.005	87.7±3.7	83.1±1.4	87.5±1.2	2.8	3.8	-0.91
Diazinon	I	305.1	169.0	14514.20	7515419.68	0.9992	0.01	0.005	105.9±4.3	91.7±2.2	86.4±2.0	4.2	3.9	-0.19
Dichlorvos	I	221.0	109.0	2452.115	1562795.08	0.9996	0.01	0.005	72.1±7.7	102.2±5.5	89.9±2.0	4.5	5.8	-4.49
Diethofencarb	F	268.1	124.0	56222.15	7750921.20	0.9964	0.01	0.005	91.3±9.7	92.2±0.8	87.5±1.6	2.6	5.5	-1.08
**Difenoconazole***	F	406.0	251.0	-3834.513	2057850.3	0.9996	1.5	0.005	100.4±11.1	93.8±1.7	86.8±3.9	2.7	17.3	350.66
Diflubenzuron	I	311.0	158.0	17352.11	3565217.34	0.9967	0.01	0.005	94.1±7.0	91.6±2.2	83.3±1.2	5.9	8.0	-0.99
Diflufenican	H	395.0	266.0	2207.146	5110187.75	0.9996	0.01	0.005	117.5±17.2	91.5±1.8	87.6±1.6	5.6	8.3	-2.36
Dimethachlor	H	256.0	224.0	86299.91	19096877.6	0.9981	0.01	0.005	85.7±8.0	95.1±0.2	91.5±1.9	1.4	5.0	0.33
Dimethoate	I	230.0	125.0	100192.4	8034508.30	0.9932	0.01	0.025	20.1±12.8	96.3±4.4	93.3±0.5	4.9	5.8	-5.52
**Dimethomorph***	F	388.0	301.1	14789.18	3071263.42	0.9975	2.00	0.005	117.9±9.6	79.6±3.4	84.5±2.6	2.6	18.3	1.76
Dimoxystrobin	F	327.1	205.1	98749.07	20167011.3	0.9978	0.01	0.005	95.7±1.0	87.2±0.1	86.3±3.3	3.6	5.4	1.19
Diniconazole	F	326.0	70.1	12046.25	1829737.76	0.9979	0.01	0.005	103.2±12.9	85.7±4.0	83.6±1.7	6.2	8.3	0.38
Dinotefuran	I	203.1	129.0	485.3208	73336.8196	0.9982		0.005	109.6±2.6	72.9±0.1	82.0±2.7	6.8	19.7	-8.78

Dioxacarb	I	224.0	167.0	49210.91	9939509.94	0.9975		0.025	32.9±6.0	96.5±2.2	89.3±1.1	3.2	5.3	-5.46
Diuron	H	233.0	72.1	76174.46	16755692.6	0.9984	0.01	0.005	70.5±3.6	93.1±1.7	92.0±1.0	1.7	3.7	-1.56
Epoxyconazole	F	330.0	121.1	24657.15	5623578.47	0.9986		0.005	96.9±1.3	89.5±1.1	86.6±4.2	1.0	8.2	0.88
Ethidimuron	H	265.1	207.9	74143.87	8194081.11	0.9950		0.025	64.1±6.5	85.0±2.4	80.3±1.1	2.4	14.6	29.35
Ethion	I	385.0	199.0	38220.04	9262611.76	0.9979	0.01	0.005	103.8±9.1	92.6±1.0	85.5±0.8	1.0	11.2	3.11
Ethirimol	F	210.1	140.1	27366.65	8655297.04	0.9986	0.01	0.005	72.8±0.9	71.0±1.9	75.3±1.3	4.6	4.8	-11.17
Ethofumesat	H	287.0	121.1	1132.710	138862.992	0.9974	0.03	0.005	96.3±19.3	85.6±8.9	88.0±2.6	8.9	17.3	7.05
Ethoprophos	I	243.0	97.0	40242.35	7816784.22	0.9981	0.01	0.025	41.8±7.4	92.4±1.4	90.1±0.8	1.4	5.9	-2.97
Etofenprox	I	394.2	177.2	62909.34	10858748.8	0.9997	0.01	0.025	16.8±11.7	98.2±4.9	92.7±1.1	4.9	27.0	1.82
Famoxadone	F	392.1	331.2	2162.304	365131.456	0.9962	0.01	0.005	85.9±17.8	94.5±1.4	84.1±5.3	1.4	8.7	3.16
Fenamidone	F	312.1	92.1	12271.67	7336700.36	0.9996	0.01	0.005	92.8±3.8	86.4±1.9	88.1±1.4	1.9	6.4	1.91
Fenamiphos	I	304.1	217.1	84498.86	13076637.4	0.9975		0.005	78.1±1.2	80.1±2.0	81.3±0.7	2.0	2.9	0.45
Fenazaquin	A	307.2	57.1	25934.35	24740591.7	0.9996	0.01	0.005	97.6±5.3	94.6±1.7	89.6±1.6	1.7	13.5	-0.84
Fenbuconazole	F	337.1	125.1	1943.278	2547769.95	0.9997	0.01	0.005	95.1±11.2	93.1±5.7	83.1±0.9	5.7	10.1	-0.03
Fenobucarb	I	208.1	95.1	19642.19	7266790.69	0.9989		0.005	93.3±3.3	88.9±1.8	92.4±2.9	1.8	8.3	-5.26
Fenoxycarb	I	302.1	88.1	48188.84	6935984.93	0.9966		0.005	96.1±1.6	91.4±3.2	88.7±0.7	3.2	6.3	-1.04
Fenpropidin	F	274.0	147.0	8756.210	8322344.23	0.9994	0.01	0.005	78.3±3.9	91.2±1.2	91.7±1.4	1.2	5.5	-4.04
Fenpyroximat	I	422.1	366.2	20610.13	8984948.49	0.9989	0.01	0.005	104.7±8.7	93.6±4.2	86.3±1.6	4.2	16.0	0.14
Fenuron	H	165.1	72.0	5005.089	856302.811	0.9963		0.025	19.1±8.0	91.5±5.3	93.1±2.7	5.3	14.4	0.52
Fipronil	I	435.0	330.0	3451.152	464225.288	0.9970		0.005	77.5±6.6	84.5±7.6	84.6±3.7	7.6	9.5	-0.55
Flonicamid	I	230.0	203.0	11638.35	1048032.25	0.9939	0.03	0.005	95.7±5.9	94.8±4.7	88.5±1.0	4.7	6.3	-0.48
Fluazinam	F	462.9	415.9	1710.622	302726.776	0.9953	0.01	0.005	99.2±3.4	70.1±1.2	72.9±2.9	2.4	12.1	-1.50
Flubendiammide	I	681.0	254.0	1496.266	77461.2386	0.9821		0.025	63.8±15.9	84.3±0.6	88.3±2.7	26.2	11.9	-4.19
Fludioxonil	F	247.0	126.0	1580.358	126008.730	0.9966	0.01	0.005	80.9±12.7	99.0±3.0	96.3±1.7	3.0	20.4	-0.07
Flufenacet	H	364.0	194.2	69624.66	10548857.2	0.9972	0.05	0.005	90.0±7.5	89.9±1.4	85.6±4.0	1.4	7.6	-1.15
Flufenoxuron	I	489.0	158.0	899.3097	1755895.24	0.9996	0.01	0.005	100.3±3.1	97.6±4.9	89.7±2.4	4.9	24.6	4.34
Flumioxazin	H	355.1	327.1	247.8841	190848.127	0.9999	0.02	0.005	85.0±17.0	87.1±9.5	82.1±4.0	9.5	13.9	-1.91
Fluometuron	H	233.1	72.1	61739.52	14219755.0	0.9985	0.01	0.005	83.0±2.9	93.2±0.5	93.2±1.5	0.5	4.5	-2.23
Fluopicolide	F	382.9	172.9	49246.99	6955173.46	0.9951	0.01	0.005	97.7±8.1	94.1±2.1	88.0±2.3	2.1	6.4	5.78
Fluoxastrobin	F	459.0	427.1	23244.93	5911796.54	0.9982	0.01	0.005	104.6±5.4	79.3±1.7	81.6±3.2	1.7	4.0	-1.74
Fluquinconazole	F	376.0	307.0	8122.275	1177218.13	0.9972	0.01	0.005	91.3±9.3	86.5±5.0	88.2±1.5	5.0	12.6	-1.17
Flusilazole	F	316.0	247.2	20447.29	4459621.57	0.9989		0.005	84.3±7.9	87.0±2.8	87.3±1.4	2.8	8.0	-1.19
Flutriafol	F	302.0	70.1	19247.25	3875268.78	0.9976	0.01	0.005	74.1±7.7	89.5±6.0	86.3±1.5	6.0	6.7	-2.30
Forchlorfenuron	H	248.1	129.0	24618.20	4696786.12	0.9979	0.01	0.005	72.4±0.7	72.6±6.9	75.8±2.1	6.9	7.6	-0.40
Fosthiazate	N	284.0	104.1	31221.20	19844745.6	0.9993	0.02	0.005	82.7±3.2	94.6±2.3	90.3±1.1	2.3	4.2	-2.64
Fuberidazol	F	185.1	157.1	29095.97	16593386.0	0.9990	0.01	0.005	104.0±8.3	81.4±3.1	85.3±1.6	3.1	5.3	-12.57
Furalaxyl	F	302.1	95.0	124343.8	27267491.1	0.9979		0.005	79.8±4.3	90.3±2.1	88.6±1.7	2.1	4.0	-0.40
Furathiocarb	I	383.1	195.1	54743.95	8372321.61	0.9971	0.00[illegible]	0.005	101.0±8.4	89.5±4.6	85.8±2.9	4.6	4.9	0.42
Halofenozide	I	329.1	120.9	6139.723	361871.260	0.9917		0.005	88.1±2.8	91.1±4.7	88.7±2.1	4.7	12.4	0.83
Hexaflumuron	I	461.0	158.0	-434.3254	440662.153	0.9975		0.005	108.9±18.7	98.5±2.7	83.4±2.2	2.7	21.6	-0.50
Hexythiazox	I	353.0	228.1	310.5641	3670278.20	0.9997	0.01	0.005	88.0±8.8	90.8±3.5	87.8±0.9	3.5	21.9	0.91
Hydramethylnon	I	495.2	323.0	-421.7070	4685207.66	0.9982		0.005	79.0±4.1	83.7±5.7	100.2±3.7	5.7	4.6	-19.22
Imazalil	F	297.0	159.0	6739.092	2029102.97	0.9993	0.01	0.005	73.8±6.4	75.7±5.8	76.7±1.7	5.8	7.7	-2.05
Imidacloprid	I	256.0	175.1	14853.33	2117597.72	0.9973	0.01	0.005	112.1±4.1	84.7±5.7	82.7±2.2	5.7	19.2	14.64
Indoxacarb	I	528.0	203.0	2397.509	591609.191	0.9988	0.2	0.005	110.8±10.4	71.6±5.2	84.5±3.3	7.6	12.2	7.16
Ipconazole	F	334.1	70.0	21072.53	5541527.38	0.9991	0.01	0.005	97.9±9.6	88.9±3.9	84.4±3.2	3.9	8.2	3.18
Iprovalicarb	F	321.2	119.1	36143.08	12917174.6	0.9979	0.01	0.005	84.3±1.2	94.6±2.5	85.6±1.4	2.5	9.7	0.65
Isocarbophos	I	231.0	121.0	78121.20	7163761.24	0.9924		0.005	109.2±18.1	89.9±3.7	87.4±0.7	3.7	5.8	-3.02
Isofenphos-methyl	I	332.0	121.0	3060.022	1096887.85	0.9993		0.005	91.5±15.9	94.5±4.1	88.8±2.7	4.1	7.1	-0.43
Isoprothiolane	F	291.1	231.0	66457.87	22278691.4	0.9991	0.01	0.005	82.9±3.5	93.5±1.6	89.8±2.1	1.6	6.9	1.15
Isoxaben	H	333.2	165.0	86988.25	19273517.7	0.9982	0.02	0.005	93.3±3.5	88.2±2.2	85.8±1.8	2.2	5.5	-0.97
Isoxaflutole	H	359.8	250.9	3547.262	669255.099	0.9968	0.02	0.005	92.7±13.4	83.4±4.9	88.8±1.8	4.9	9.5	-2.23
Ivermectin B1a	N	892.5	350.8	63.51286	9505.13815	0.9936		0.005	107.4±11.6	78.6±12.3	72.5±9.5	12.3	12.5	-0.29
Kresoxim-methyl	F	314.1	267.1	11771.14	2420600.22	0.9986	0.01	0.005	93.3±6.1	90.7±3.0	90.1±0.2	3.0	12.5	-1.17
Lenacil	H	235.2	153.1	25806.71	5941856.73	0.9985	0.1	0.005	80.7±1.4	87.2±4.8	76.8±1.6	4.8	7.5	11.85
Linuron	H	249.0	160.0	14959.50	3031511.67	0.9977	0.01	0.005	92.6±1.6	91.6±2.6	90.2±1.7	2.6	5.2	0.48
Lufenuron	I	510.9	158.0	2012.082	480791.709	0.9979	0.01	0.005	70.9±13.2	88.0±4.8	85.9±1.1	4.8	19.4	-0.81
Malaoxon	I	215.1	99.0	23842.08	15298120.2	0.9991	0.02	0.005	76.5±1.8	94.8±1.8	87.6±1.5	1.8	5.4	1.72
Malathion	I	331.0	126.9	17439.58	3598062.19	0.9965	0.02	0.005	88.9±2.9	95.5±5.1	88.2±1.8	5.1	6.5	-2.33
**Mandipropamid***	F	411.9	328.1	18427.82	5601162.80	0.9989	0.3	0.005	89.2±4.0	80.8±4.6	82.4±2.9	4.6	9.4	-0.34
Mecarbam	I/A	330.0	97.1	57366.61	6545906.18	0.9961	0.01	0.005	92.2±6.0	86.3±4.9	89.0±1.3	4.9	7.7	0.28
Mepanipyrim	F	224.0	106.1	29648.26	4444632.75	0.9967	0.01	0.005	90.2±0.8	91.1±2.7	89.7±2.6	2.7	9.7	3.63

Metaflumizone	I	507.0	178.1	1043.167	168825.643	0.9968	0.02	0.005	96.5±17.9	96.1±16.0	90.3±2.3	16.0	14.5	5.46
**Metalaxyl-M***	F	280.1	220.2	56937.90	16038149.3	0.9993	0.05	0.005	93.8±4.8	90.2±3.3	90.5±2.2	3.3	4.2	2.33
Metamitron	H	203.1	175.1	19830.38	2632360.79	0.9965	0.01	0.005	86.0±10.1	91.7±3.0	90.7±0.9	3.0	6.3	-8.05
**Metazachlor***	H	278.0	134.1	10631.60	16118959.5	0.9997	0.06	0.005	86.2±3.1	94.0±1.1	89.7±2.0	1.1	3.7	2.26
Metconazole	H	320.1	70.1	23407.85	4831724.84	0.9982	0.02	0.005	95.1±10.4	85.4±1.7	83.3±1.7	1.7	8.2	-0.16
Methabenzthiazuron	F	222.0	165.0	64494.56	23926399.4	0.9990	0.01	0.005	72.1±4.0	89.1±2.4	88.6±1.7	2.4	5.2	-2.86
Methacrifos	H	241.0	125.1	1993.875	812325.989	0.9990	0.01	0.005	81.7±3.4	93.2±7.3	89.3±1.7	7.3	14.8	1.53
Methamidophos	I	141.9	94.1	145.7735	8520825.26	0.9996	0.01	0.005	94.5±3.6	81.6±8.2	78.5±2.9	8.2	10.3	-0.14
Methidathion	I	302.9	85.1	24873.58	2948746.41	0.9952	0.02	0.005	98.1±2.3	91.6±1.6	90.1±2.2	1.6	8.2	-1.74
Methiocarb	I	226.1	121.1	37877.12	6359291.88	0.9968	0.03	0.005	94.8±6.5	88.2±2.4	88.0±1.8	2.4	10.9	-3.39
Methomyl	I	162.9	106.1	58539.28	9505990.10	0.9969	0.01	0.005	110.0±0.5	119.3±5.8	110.8±0.8	5.8	5.7	-1.19
Methoprotryne	H	272.2	198.0	33470.60	12190017.7	0.9986		0.005	82.9±1.0	93.5±1.5	88.3±2.9	1.5	2.7	-0.50
Methoxyfenozide	I	369.2	149.1	56166.75	9755113.19	0.9978	0.01	0.005	87.8±6.6	84.9±1.9	86.5±3.7	1.9	8.1	0.07
Metobromuron	H	259.0	170.0	8111.274	2517705.64	0.9979		0.005	94.5±4.9	91.9±1.5	91.0±1.7	1.5	8.8	-1.03
Metolachlor	H	284.1	252.2	61872.64	18818835.3	0.9991	0.05	0.005	98.2±1.1	95.9±1.9	88.8±0.9	1.9	4.7	1.33
Metrafenon	F	409.0	209.1	22601.58	4379471.24	0.9983	0.01	0.005	100.3±10.1	91.2±5.0	90.1±2.7	5.0	9.4	-0.48
Metribuzin	H	215.0	187.1	4806.053	2117019.48	0.9993	0.1	0.025	57.6±12.2	89.7±2.1	90.7±0.8	2.1	7.5	-3.03
Mevinphos	I	225.0	127.0	17839.89	3598593.56	0.9975	0.01	0.005	85.4±10.2	95.7±4.7	87.4±1.6	4.7	3.7	-0.77
Mexacarbate	I	223.1	151.0	24996.56	25441441.8	0.9998		0.005	77.0±2.2	85.7±3.6	80.4±1.3	3.6	3.8	-3.98
Molinate	A	188.1	126.0	1228.071	1242322.93	0.9996	0.01	0.005	105.8±4.7	91.3±3.4	88.5±3.5	3.4	3.6	-1.00
Monocrotophos	I	224.0	127.0	2465.809	3876358.64	0.9998	0.01	0.005	79.6±1.0	92.2±6.6	85.9±2.3	6.6	7.0	-3.08
Moxidectin	I	640.4	528.2	316.4565	43474.1664	0.9925		0.005	109.2±7.0	100.4±8.6	78.3±3.2	8.6	18.7	-8.97
**Myclobutanil***	F	289.1	70.1	16822.31	3447137.65	0.9984	0.8	0.005	111.4±2.9	85.1±3.5	88.3±0.5	3.5	5.3	0.79
Nitenpyram	I	271.1	56.1	5696.981	2970621.56	0.9994		0.025	67.8±12.2	78.1±6.0	79.7±1.2	6.0	6.4	-6.42
Novaluron	I	493.1	158.1	-1971.241	1116735.66	0.9997	0.01	0.005	92.1±15.9	90.1±11.0	87.5±1.7	11.0	16.4	2.75
Omethoate	I	214.0	125.0	12924.26	12519633.2	0.9996	0.01	0.005	94.4±2.9	86.0±5.2	83.5±1.2	5.2	3.8	1.48
Oxadiazon	H	345.0	303.0	1292.954	698034.088	0.9981	0.01	0.005	88.2±2.5	90.2±2.9	82.5±4.5	2.9	17.2	4.53
Oxadixyl	F	279.1	219.1	2447.631	5518158.11	0.9995	0.01	0.005	85.3±5.3	94.8±8.7	88.4±0.8	8.7	18.1	-55.32
Oxamyl	I	237.0	72.1	20204.07	18182380.7	0.9996	0.01	0.005	83.0±1.9	90.7±5.0	87.5±1.7	5.0	4.8	-2.02
Paclobutrazol	F	294.1	70.1	46458.28	6309048.40	0.9960	0.01	0.005	89.0±1.2	86.7±0.9	83.8±2.1	0.9	10.6	-0.01
**Penconazole***	F	284.0	70.1	44861.34	4659759.99	0.9951	0.06	0.005	90.8±7.8	87.9±3.8	87.3±0.6	3.8	6.6	-0.09
Pencycuron	F	329.1	125.0	56718.71	14661786.9	0.9997	0.02	0.005	96.6±6.3	97.3±2.5	100.2±2.9	2.5	5.8	0.25
**Pendimethalin***	H	282.1	212.1	3756.808	2230600.54	0.9997	0.05	0.005	102.0±2.9	92.3±2.2	90.6±1.6	2.2	17.0	3.55
Phenmedipham	H	318.1	136.0	-5725.130	11372050.3	0.9958	0.01	0.005	82.7±6.2	82.6±5.4	85.3±1.0	5.4	19.4	-0.96
Phenthoate	I	321.0	79.1	54241.59	6203238.95	0.9958		0.005	100.6±1.5	93.5±4.5	90.9±2.6	4.5	10.6	1.16
Phosalone	I	368.0	182.0	8473.808	1789802.78	0.9956	0.01	0.005	110.7±10.8	92.7±2.5	81.5±0.8	2.5	10.7	5.73
Phosmet	I	317.9	169.0	41686.90	9213612.80	0.9977	0.05	0.005	87.9±1.7	86.2±1.8	86.2±4.1	1.8	10.4	-0.78
Phosphamidon	I	300.1	174.1	-2230.300	5775658.86	0.9997	0.01	0.005	84.1±3.9	93.9±3.4	89.7±0.7	3.4	23.8	-2.33
Phoxim	I	299.1	77.1	40045.71	7182618.11	0.9962	0.01	0.005	97.6±6.7	90.2±4.2	87.3±0.3	4.2	7.9	-2.47
Picolinafen	H	377.1	238.0	53247.29	8206120.89	0.9959	0.01	0.005	105.1±14.5	90.8±1.6	85.6±2.0	1.6	10.7	2.31
Picoxystrobin	F	368.1	145.1	73794.72	22252110.3	0.9990	0.01	0.005	97.1±5.3	89.3±0.9	87.0±3.3	0.9	6.2	-2.63
**Pirimicarb***	I	239.1	72.1	23246.21	27923650.9	0.9995	5.00	0.025	66.3±2.3	93.3±2.7	88.8±1.0	2.7	4.7	-0.26
Pirimiphos-methyl	I	306.0	164.2	15709.38	7289446.00	0.9993	0.01	0.005	84.3±4.8	95.7±1.9	89.1±3.6	1.9	7.8	-0.19
Prochloraz	F	376.0	308.0	33434.97	4613380.72	0.9961	0.03	0.005	82.2±6.7	70.1±1.9	73.9±3.0	1.9	7.7	-2.91
Profenofos	I	374.9	304.9	9855.093	3652199.30	0.9990	0.01	0.005	109.4±8.6	91.8±3.5	86.4±2.0	3.5	7.9	2.65
Promecarb	I	208.1	109.1	20455.91	7736053.12	0.9991		0.005	89.9±3.5	95.2±0.7	92.1±1.5	0.7	10.6	-0.33
Prometon	H	226.2	142.1	33355.67	19027181.6	0.9992		0.005	85.6±3.0	92.1±3.6	88.8±0.5	3.6	4.0	-1.89
Propamocarb	F	189.1	102.1	-83318.63	30883726.4	0.9999	0.01	0.005	75.9±2.9	81.0±5.6	78.6±1.0	5.6	4.8	13.97
Propaquizafop	H	444.0	100.2	13517.15	5065526.40	0.9992	0.01	0.005	79.0±1.6	72.5±2.2	70.1±0.8	4.9	9.2	2.44
Propargite	I	368.1	321.2	35135.95	8909959.10	0.9977	0.01	0.005	104.1±15.2	95.5±2.0	88.3±1.5	2.0	13.2	-1.79
Propetamophos	I	282.1	138.0	9374.888	1247635.43	0.9981		0.005	99.1±14.1	86.6±4.8	91.7±5.2	4.8	13.2	7.19
Propham	H	180.1	138.1	-2394.587	1877062.96	0.9998	0.01	0.005	99.0±2.2	93.8±8.3	90.5±2.0	8.3	5.9	0.16
Propiconazole	F	342.0	158.9	4437.237	1587857.70	0.9960	0.01	0.005	118.0±11.2	100.0±18.3	83.8±2.2	18.3	19.7	4.94
Propoxur	I	210.1	111.1	17378.35	15809116.0	0.9998	0.05	0.005	69.2±0.5	95.9±2.9	92.9±2.2	2.9	5.4	-1.66
**Propyzamide***	H	294.1	70.1	33145.94	3894242.04	0.9967	0.02	0.005	112.7±10.6	92.2±4.6	91.2±3.0	4.6	12.2	5.22
Proquinazid	F	372.9	289.0	4822.904	4194664.16	0.9998	0.01	0.005	95.3±1.6	98.2±3.2	90.3±2.9	3.2	17.4	2.75
Prosulfocarb	H	252.1	91.1	29084.83	20106836.1	0.9997	0.01	0.005	101.1±6.5	95.4±3.2	90.4±1.1	3.2	7.8	-1.71
Pyracarbolid	F	218.1	125.0	60299.97	26509712.6	0.9993		0.005	81.8±4.9	91.4±1.1	90.7±1.5	1.1	4.8	-1.72
**Pyraclostrobin***	F	388.0	163.1	17258.91	6163673.15	0.9993	3	0.005	104.0±2.8	91.3±3.2	91.8±1.1	3.2	4.3	-1.18
Pyridaben	A	365.1	147.1	39965.31	17664739.7	0.9992	0.01	0.005	107.6±19.6	94.7±3.1	90.1±1.3	3.1	13.1	-1.49
Pyrimethanil	F	200.1	82.1	3086.545	2106173.46	0.9997	0.01	0.005	85.5±2.2	83.8±4.3	90.2±1.0	4.3	7.9	-0.17
Pyriproxyfen	I	322.1	96.1	52267.87	17202339.4	0.9990	0.05	0.005	92.7±3.2	91.9±2.9	91.0±2.3	2.9	9.4	-3.97

Quinalphos	I	299.0	163.0	19040.66	2423278.27	0.9958	0.01	0.005	104.0±10.5	92.9±5.7	89.7±3.1	5.7	6.0	5.45
Quinoclamin	H	208.0	76.9	6321.536	2060652.22	0.9988	0.01	0.005	71.4±11.9	108.2±2.7	109.3±2.1	2.7	10.1	-18.24
Quinoxyfen	F	308.0	197.0	10080.47	3245774.93	0.9984	0.3	0.005	94.0±10.1	99.2±1.4	90.9±1.7	1.4	10.3	2.30
**Quizalofop-ethyl***	H	373.1	271.2	906.1421	641103.116	0.9994	0.01	0.005	90.0±5.2	102.9±2.0	91.5±2.1	2.0	19.0	4.20
Rotenone	I	395.0	213.1	-941.1177	1207886.54	0.9997	0.01	0.005	97.3±10.2	84.2±1.9	87.1±2.5	1.9	8.0	2.63
Secbumeton	H	226.2	170.1	32710.64	16692153.8	0.9992		0.005	85.7±1.3	91.7±3.8	89.4±1.4	3.8	4.1	-1.56
Silthiopham	F	268.0	252.1	60629.11	5964111.58	0.9948		0.005	89.2±3.9	90.8±3.9	89.3±1.2	3.9	5.6	-2.74
**Spinosyn A***	I	732.4	142.1	5787.656	4027824.43	0.9997	0.15	0.005	93.6±3.3	78.9±3.6	85.9±1.7	3.6	5.2	-5.16
**Spinosyn D***	I	746.5	142.1	1220.199	641794.778	0.9987	0.15	0.005	96.4±4.5	85.1±5.7	83.5±3.4	5.7	16.8	-6.35
Spirodiclofen	I	411.1	71.2	10350.59	3013415.27	0.9974	0.02	0.005	100.9±10.3	84.0±7.5	78.2±2.9	7.5	12.5	-1.03
Spiromesifen	I	388.2	273.0	7374.867	4367402.70	0.9995	0.02	0.005	114.4±2.4	85.6±5.7	81.1±11.7	5.7	13.8	-0.39
**Spirotetramat***	I	374.1	330.3	6752.596	2035996.12	0.9997	1.00	0.005	97.1±6.6	70.6±5.9	82.0±2.9	9.9	8.4	-0.87
Spiroxamine	F	298.2	144.2	13409.14	10130816.7	0.9994	0.01	0.005	93.1±1.4	92.7±2.9	89.9±1.8	2.9	6.8	-2.07
**Tebuconazole***	F	308.1	70.1	33272.37	4563920.94	0.9966	0.6	0.005	114.5±8.8	90.5±5.0	88.1±1.6	5.0	7.1	-1.58
Tebufenozid	H	353.0	133.1	79425.45	11530422.5	0.9967	0.01	0.005	83.6±4.8	85.6±3.6	86.5±1.6	3.6	9.3	-2.40
Tebufenpyrad	I	334.1	117.1	11413.28	3143109.88	0.9992	0.01	0.005	103.2±22.8	89.3±1.7	88.4±0.2	1.7	11.8	3.60
Tebuthiuron	I	229.1	172.1	64466.81	17559462.5	0.9988		0.005	80.4±3.9	90.7±3.6	87.7±1.5	3.6	3.8	-1.84
Teflubenzuron	H	379.0	339.0	4330.323	138946.019	0.9787	0.01	0.005	78.8±9.1	87.9±2.1	94.7±2.5	2.1	7.6	-10.16
Temephos	I	467.0	124.9	6106.597	2016027.27	0.9984		0.005	110.6±4.6	88.8±7.7	87.9±2.9	7.7	10.7	1.23
**Tetraconazole***	F	372.0	159.0	11225.79	1419234.74	0.9969	0.2	0.005	115.0±8.3	90.3±5.7	89.6±1.5	5.7	9.6	-0.63
Thiabendazole	F	202.0	175.1	-556.2830	17269977.6	0.9998	0.01	0.005	71.6±1.6	81.9±2.6	79.5±0.5	2.6	5.5	-3.35
Thiacloprid	I	253.0	126.0	96982.10	15444680.4	0.9972	0.01	0.005	71.7±1.8	92.9±3.1	91.2±1.1	3.1	5.8	-1.30
Thiamethoxam	I	292.0	211.1	25556.04	3355902.94	0.9968	0.5	0.005	118.7±0.2	90.0±5.3	87.9±0.9	5.3	8.3	2.54
Thidiazuron	H	221.0	101.9	4223.751	1497690.01	0.9991		0.005	93.5±1.4	81.7±0.4	81.9±2.1	0.6	9.5	-12.02
Thiodicarb	I	355.0	88.1	27461.50	5629973.55	0.9975	0.01	0.005	83.7±8.0	76.4±1.9	77.8±4.6	1.9	4.7	-2.25
Thiofanox	I	241.1	184.1	592.2083	98440.9227	0.9989		0.025	54.4±8.2	76.6±7.7	91.0±6.4	7.7	17.9	-6.77
Tolclofos-methyl	F	300.9	125.0	1152.983	700533.274	0.9989	0.01	0.005	111.5±12.4	95.9±3.3	88.0±2.7	3.3	15.8	-2.70
Triadimefon	F	294.1	69.1	4256.620	2996496.33	0.9989	0.01	0.005	112.2±19.5	94.5±4.7	86.9±2.3	4.7	19.7	-5.14
Triadimenol	F	296.1	70.1	5593.257	989691.627	0.9986	0.70	0.005	86.5±5.5	85.5±1.2	89.8±2.1	1.2	13.0	3.01
Triazophos	I	314.0	162.1	1231.879	129428.610	0.9854	0.01	0.005	121.6±29.3	118.4±14.0	78.7±5.0	14.0	21.6	5.85
Trichlorfon	I	256.9	221.0	2629.580	331030.563	0.9880	0.01	0.005	75.5±5.0	82.3±15.3	80.3±2.3	15.3	11.8	-2.35
Tricyclazol	F	190.0	136.0	122861.7	14476873.7	0.9962	0.01	0.005	72.7±1.5	83.1±2.6	83.6±1.4	2.6	5.1	0.58
Trietazin	H	230.1	99.0	29879.20	4266908.45	0.9968		0.005	94.5±7.5	91.8±3.2	91.2±2.0	3.2	9.5	-0.72
Trifloxystrobin	F	409.1	186.1	21366.50	11840608.4	0.9991	0.30	0.005	91.5±3.8	88.3±4.0	87.5±1.3	4.0	7.1	-4.92
Triflumuron	I	359.0	156.0	24770.82	2447749.47	0.9962	0.01	0.005	108.8±4.9	83.4±5.1	91.3±1.4	5.1	8.4	-0.65
Trimethacarb	I	194.1	137.0	82201.87	14216097.8	0.9986		0.005	89.3±4.3	93.3±2.8	93.7±0.7	2.8	7.4	-0.94
Triticonazole	F	318.1	70.1	8339.098	2492676.82	0.9992	0.01	0.005	98.7±5.6	87.1±7.5	84.0±3.0	7.5	7.9	-2.16
Uniconazole-P	H	292.1	70.0	14143.89	3278139.08	0.9979		0.005	90.3±8.7	88.7±3.9	86.6±1.0	3.9	15.0	-0.99
Vamidothion	I	288.1	146.0	14659.05	20679629.5	0.9997		0.025	50.6±2.6	84.5±4.2	80.4±1.2	4.2	4.7	-3.12
Zoxamide	F	336.0	187.0	23983.51	7588401.84	0.9994	0.02	0.005	97.8±6.8	92.3±5.0	91.7±1.5	5.0	7.8	2.37

*Pesticide authorized in Italy on artichoke

y I: Insecticide; F: Fungicide; H: herbicide; A: Acaricide; N: Nematocide;
